# Cardiac 123I-MIBG Scintigraphy in Neurodegenerative Parkinson Syndromes: Performance and Pitfalls in Clinical Practice

**DOI:** 10.3389/fneur.2019.00152

**Published:** 2019-02-26

**Authors:** Cornelia Skowronek, Leonora Zange, Axel Lipp

**Affiliations:** ^1^Movement Disorders and Neuromodulation Section, Charité - Universitätsmedizin Berlin, corporate member of Freie Universität Berlin, Humboldt-Universität zu Berlin, and Berlin Institute of Health, Berlin, Germany; ^2^Berlin Institute of Health, Berlin, Germany; ^3^Working Group on Cardiovascular Magnetic Resonance, Department of Cardiology and Nephrology, Experimental and Clinical Research Center, Charité Medical Faculty and the Max-Delbrück Center for Molecular Medicine and HELIOS Hospital Berlin Buch, Berlin, Germany; ^4^Department of Neurology, Park-Klinik Weissensee, Berlin, Germany

**Keywords:** Parkinson‘s disease, Lewy body disorders, multiple system atrophy, MIBG scintigraphy, autonomic function

## Abstract

**Purpose:** Cardiac [^123^I]metaiodobenzylguanidine scintigraphy (123I-MIBG), reflecting postganglionic cardiac autonomic denervation, is proposed for early detection of Parkinson's disease (PD; reduced tracer uptake) and separation from Multiple System Atrophy (MSA; preserved tracer uptake). However, several recent studies report on frequent unexpected 123I-MIBG results in PD and MSA. We sought to determine, whether 123I-MIBG is feasible to discriminate PD from MSA in unselected geriatric patients in clinical practice.

**Materials and Methods:** We screened consecutive patients, that underwent 123I-MIBG for diagnostic reasons. Delayed 123I-MIBG uptake (heart/mediastinum ratio; H/M ratio) was verified by clinical diagnosis of PD, MSA, and ET based on a two-stage clinical assessment: comprehensive baseline (including autonomic testing and additional neuroimaging) and confirmatory clinical follow-up.

**Results:** 28 patients with clinical diagnosis of PD (*N* = 11), MSA (*N* = 9), and Essential Tremor (ET, *N* = 8) were identified. In one third (9/28) nuclear medical diagnosis deviated from clinically suspected syndrome. Visual interpretation of 123I-MIBG identified two cases (MSA and ET) with indeed normal 123I-MIBG uptake. Detailed review of clinical phenotypes provided only in two cases (PD and ET) an adequate explanation (correction of initial diagnosis and confounding drug history) for unexpected 123I-MIBG. In conclusion, 123I-MIBG did not match initial clinical phenotype in 27% PD, 44% MSA, and 25% ET patients.

**Conclusion:** 123I-MIBG scintigraphy is a known specific and valuable technique in scientific approaches and well-defined and highly selected samples. However, predictability of 123I-MIBG based nuclear medical diagnosis for individual cases and thus, feasibility in routine clinical practice is limited. Our clinical series emphasize clinical verification of 123I-MIBG results on an individual basis in clinical routine.

## Introduction

Parkinson's Disease (PD) and Multiple System Atrophy (MSA) are pathophysiological distinct disorders, that share a common clinical phenomenology. When compared to PD, MSA is characterized by a more rapid deterioration and limited clinical response to levodopa substitution. Despite the use of clinical consensus criteria (1, 2) discrimination of MSA and PD, especially at early stages, is challenging as neuropathological confirmed case series indicate (3).

Cardiac 123I-metaiodobenzylguanidine (123I-MIBG) scintigraphy is used to discriminate PD and MSA by means of cardiac postganglionic autonomic involvement (4–6). Cardiac uptake of the synthetic norepinephrine analog (123I-MIBG) depends on integrity of postganglionic sympathetic neurons. Since α-synuclein dependent neurodegeneration in PD affects both pre- and postganglionic autonomic neurons, cardiac 123I-MIBG uptake is impaired, whereas in MSA, with predominately preganglionic autonomic failure, cardiac 123I-MIBG uptake is thought to be preserved. Braune et al. (6) reported on 100% sensitivity and specificity of cardiac 123I-MIBG scintigraphy in differentiating PD and MSA, respectively. Recent clinical studies confirmed the high sensitivity and report on specificity more than 77% (7, 8). As such, 123I-MIBG scintigraphy is recommended (level A) by the European Federation of Neurological Societies and the Movement Disorder Society task force (9) for the differential diagnosis of Parkinson syndromes.

However, there is an increasing number of reports on impaired cardiac autonomic innervation in non-idiopathic Parkinson such as MSA. In a longitudinal study, Nagayama et al. (10) report consistently on a high sensitivity (87.7%) of cardiac 123I-MIBG scintigraphy in PD, but 179 of 269 patients (66.5%) with non-Lewy body pathology showed a diminished cardiac 123I-MIBG uptake as well, resulting in a low specificity of 37.4%. In recent studies up to 30% of MSA patients present with reduced cardiac 123I-MIBG uptake (10–12) and in one study (13) H/M ratio was diminished in even 7 out of 9 MSA patients.

These unexpected 123I-MIBG results in MSA can be attributed in part to known confounders of 123I-MIBG uptake such as myocardial lesions, cardiomyopathy, chronic heart failure or peripheral neuropathy, and concomitant medication (14). In MSA, a transsynaptic neurodegeneration causing secondary postganglionic neuronal decline is further postulated (11).

In contrast to prospective trials of highly selected samples, we report on our experience of 123I-MIBG performance in a non-selected clinical sample of patients suffering various neurodegenerative Parkinson's syndromes. We therefore verified the nuclear medical based classification of MSA, ET, and PD by combining the diagnostic value of additional neuroimaging [e.g., 123I-2β-carbomethoxy-3β-(4-iodophenyl)-N-(3-fluoropropyl) nortropane single photon emission computed tomography (123I-FP-CIT SPECT)], levodopa responsiveness, autonomic function tests and a two-stage clinical assessment covering a mean follow-up of 3 years. In this clinical series we sought to highlight potential pitfalls of cardiac 123I-MIBG imaging in routine clinical use and to discuss overestimated clinical implications.

## Patients and Methods

### Patients

Consecutive patients, that underwent 123I-MIBG scintigraphy for differential diagnosis of a Parkinson syndrome within a 24-month recruitment period at our University Medical Center, were approached to participate in this clinical series. The study was approved by the Institutional Review Board of Charité—University Medicine Berlin, Germany and all patients gave written informed consent before participation.

### Clinical Assessment

Nuclear medical diagnosis derived from the results of cardiac 123I-MIBG scintigraphy (PD: reduced 123I-MIBG uptake, MSA: preserved 123I-MIBG uptake, Essential Tremor (ET): preserved 123I-MIBG uptake). Clinical diagnosis derived from (1) a comprehensive clinical assessment based on consensus diagnostic criteria (1, 2) and appropriate scales of clinical severity [PD: UPDRS (15), MSA: UMSARS (16)], (2) standardized test of levodopa responsiveness (UPDRS motor part before and 30 min after oral administration of 200 mg levodopa/50 mg benserazide), (3) autonomic reflex screen (ARS), and (4) results of supporting imaging including transcranial mesencephalic sonography, structural MRI, dopamine transporter SPECT (123I-FP-CIT SPECT), and dopamine receptor SPECT (123I-IBZM SPECT). Clinical diagnosis was further verified by a long-term follow-up were initial diagnostic classification was re-evaluated based on progression of motor and non-motor symptoms, sustained levodopa responsiveness, and survival period. If subjects were unable to present for clinical follow-up, general practitioner or family members were contacted.

### Cardiac 123I-MIBG Scintigraphy

Cardiac scintigraphy was performed according to standard operation procedures of the Department of Nuclear Medicine, Charité—University Medicine Berlin (17). Thyroid was blocked by sodium perchlorate and drugs known to affect 123I-MIBG binding, such as α-blockers, reserpine derivates, and sympathomimetics, were stopped for least 24 h. 185 MBq 123I-MIBG (AdreView Iobenguane (123I) Injection, GE Healthcare, Braunschweig, Germany) was intravenously applied. Tracer uptake was detected by double-head gamma camera equipped with a low energy high resolution (LEHR) collimator (Millenium VG5 Hawkeye with VPC-45K collimator; GE Medical Systems-EU, Buc, France or Symbia TruePoint SPECT-CT; Siemens, Erlangen, Germany). For imaging a 15% window was focused on 159 keV. Planar anterior images were obtained 4 h (delayed) post-injection (to assess only the active neuronal tracer uptake) and analyzed using Brain Registration and Analysis Software Suites (BRASS) (Hermes Medical Solutions; Stockholm, Sweden). Regions of interest (ROI [counts/voxel]) were placed manually on planar anterior images rectangular in the upper mediastinum and circular covering the left ventricle of the heart. Delayed heart to mediastinum ratio (H/M ratio) was calculated as ROI heart/ROI mediastinum with a site-specific cut-off value of 1.7. Semi-quantitative BRASS analysis was supplemented by secondary visual interpretation. Internal control was performed by evaluation of control tissue (salivary gland) and SPECT images.

### Autonomic Reflex Screen (ARS)

Cardiovagal and adrenergic function was assessed as heart rate response to deep breathing and blood pressure response to Valsalva maneuver and passive head-up tilt, respectively (17) and scored on the objective Composite Autonomic Scoring Scale (CASS, cardiovagal, and adrenergic subscores without sudomotor function) (18).

### Statistics

Group data are expressed as mean (standard deviation; SD). Statistical significance was considered at a *p*-value < 0.05 (Prism 5.02 for Windows, GraphPad Software, Inc., CA, USA).

## Results

Within a 24-month period, we identified 28 patients (10 female, 18 male; mean age 65.5 ± 10.1 years) who underwent cardiac 123I-MIBG scintigraphy. Clinical indication for nuclear imaging were suspected Parkinson's disease (PD, *N* = 11), MSA (*N* = 9), and ET (*N* = 8) (see Table 1). Mean cardiac 123I-MIBG H/M ratio (cut-off value 1.7) of both, PD and MSA, were significantly decreased compared to patients without an underlying neurodegenerative disorder (ET 2.2 ± 0.5; MSA 1.7 ± 0.3; *p* < 0.05; PD 1.5 ± 0.5; *p* < 0.01). In this small sample however, no significance could be revealed between PD and MSA patients.

**Table 1:** Patient characteristics—epidemiologic distribution, clinical data, and neuroimaging results.

		**Controls**	**MSA**	**PD**	***p*-value**
**DEMOGRAPHIC DATA**					
*N*		8	9	11	
Age	[Years]	64.8 ± 10.5	61.1 ± 10.8	69.6 ± 8.3	0.302^a^
Gender	[f: m]	3: 5	3: 6	4: 7	
BMI	[kg/m^2^]	25.7 ± 4.1	23.0 ± 3.2	25.3 ± 4.7	0.373^a^
Disease duration	[Years]	n.a.	5.2 ± 2.6	5.5 ± 4.3	0.867^c^
UxxRS_off_	[Points]	n.a.	34.3 ± 6.1	24.3 ± 8.9	
Follow-up	[Months]	41.2 ± 18.8	37.3 ± 23.3	29.1 ± 20.8	
**AUTONOMIC FUNCTION TESTS**					
CASS_cardiovagal_	[Points]	0.1 ± 0.4	0.8 ± 1.3	0.6 ± 1.0	0.579^a^
CASS_adrenergic_	[Points]	0.7 ± 0.5	2.4 ± 1.2^d^	1.0 ± 0.0^e^	0.001^a^
ΔSBP	[mmHg]	7.1 ± 13.7	−37.4 ± 28.5^d^	−1.6 ± 14.4^e^	0.001^a^
ASP	[Points]	29.5 ± 26.3	49.0 ± 23.7	21.9 ± 14.8	0.033^b^
**NEUROIMAGING**					
H/M ratio_delayed_	n.a.	2.2 ± 0.5	1.7 ± 0.3^f^	1.5 ± 0.5^d^	<0.01^b^^,^ ^d^
SR_contralateralputamen_	n.a.	2.3 ± 0.3	0.7 ± 0.6^g^	0.7 ± 0.4^d^	<0.001^a^^,^ ^e^

*BMI, Body Mass Index; UxxRS_off_ for PD: UPDRS, Motor Impairment Score part III (off medication); UxxRS_off_ for MSA: UMSARS, Motor Impairment Score part II (off medication); CASS, adrenergic/cardiovagal subscore of Composite Autonomic Scoring Scale; ΔSBP, Change of systolic blood pressure during head-up tilt; ASP, Autonomic Symptome Profile; H/M ratio_delayed_, heart/mediastinum ratio (4 h post-injection); SR_contralateralputamen_, specific binding ratio (contralateral to the clinically more affected side), n.a., not available. p-value < 0.05 was considered to be significant*.

a *Kruskal-Wallis Test*;

b *One-way analysis of variance*;

c Unpaired, two-tailed t-test. post hoc analysis between groups (Dunn‘s Multiple Comparison Test and Tukey’s Multiple Comparison Test, respectively):

d *p < 0.01 vs. ET*;

e *p < 0.05 vs. MSA*;

f *p < 0.05 vs. ET*;

g p < 0.001 vs. ET.

Nuclear medical diagnosis deviated from clinically suspected syndrome in 9 of the 28 cases: cardiac 123I-MIBG binding was preserved in 3 PD but reduced in 4 MSA and 2 ET patients. To exclude the potential confounding effect of different reporting physicians, all 123I-MIBG scintigraphy images were re-evaluated by an experienced physician blinded for the suspected clinical diagnosis. Increased mediastinal background activity led to an adjustment of nuclear medical diagnosis in two cases with indeed normal myocardial uptake but “pathological” H/M ratio (ET H/M ratio of 1.58; MSA H/M ratio of 1.65).

The clinical phenotype, long-term follow-up, and results of additional imaging studies are discussed for the remaining 7 cases with unexpected cardiac 123I-MIBG scintigraphy results (see Table 2; detailed case description is provided as Supplementary Materials).

**Table 2:** Summary of pitfalls in cases with an unexpected 123I-MIBG scintigraphy result.

**Unexpected 123I-MIBG results**			
**Pitfall**		**Case**	**Reference**
**FALSE POSITIVE H/M RATIO**			
Autonomic Neuropathy/Ganglionopathy	Ganglionopathy	No. 3	(4)
	Diabetes mellitus	No. 4	(19)
	Post-polio syndrome	No. 2	(20)
Medication	Amiodarone	No. 4	(14)
Cardiac rhythm disease	Sick-sinus-syndrome	No. 4	(21)
**FALSE NEGATIVE H/M RATIO**			
Medication	Calcium channel blocker	No. 6	(14)
	Amiodarone (long-term administration)	No. 4	(14)
Incorrect diagnosis	Progressive Supranuclear Palsy (PSP)	No. 5	
Erroneous position of mediastinal ROI	Abnormal anatomy (post-operative/hereditary)	No. 7	

H/M ratio, heart/mediastinum ratio; ROI, region of interest.

### Case 1: 59–62 Years, Possible MSA-P

#### Clinical Phenotype

Five years of symmetric bradykinesia and rigidity with postural instability and shuffling gait; startle myoclonus and pyramidal tract signs (hyperreflexia) within left upper extremity; *non-motor*: severe palilalia and dysphagia, urge incontinence, obstipation, erectile dysfunction, sialorrhea, and mild neurogenic orthostatic hypotension; UMSARS = 38 pts.

#### Imaging

*cardiac 123I-MIBG scintigraphy:* H/M ratio 1.27.

*123I-FP-CIT SPECT:* visually asymmetric (right) reduced putaminal tracer uptake.

#### Discussion

Clinical follow up (rapid progression, early death) strengthened the initial diagnosis of MSA-P. Medical history and clinical exam did not reveal clinically significant neuropathy nor concomitant medication sufficiently explaining reduced cardiac 123I-MIBG uptake.

### Case 2: 63–66 Years, Possible MSA-C

#### Clinical Phenotype

Five years of right sided Parkinsonism (bradykinesia, rigidity), cerebellar, and pyramidal tract signs; *non-motor*: apraxia, dysphagia, obstipation, erectile dysfunction, sialorrhea, and mild neurogenic orthostatic hypotension; UMSARS = 33 pts.

#### Imaging

*cardiac 123I-MIBG scintigraphy:* H/M ratio 1.14.

*123I-FP-CIT SPECT:* visually symmetric reduced tracer uptake (putamen, striatum, nucleus caudatus).

#### Discussion

Clinical diagnosis in this case is less robust as current consensus diagnostic criteria consider dementia as a non-supporting feature of MSA. However, MODIMSA study (22) showed evidence that frontal-executive dysfunction and cognitive impairment is indeed associated with MSA. Spinocerebellar atrophy (SCA) causes progressive ataxia, Parkinsonism and pyramidal tract signs but patients' familiar history was unremarkable and genetic screen for SCA 1,2,3, and 6 was negative. Patient had a history of poliomyelitis during childhood and thus might have developed post-polio syndrome. However, lower motoneuron disability in our patient did not progress clinically nor does electromyography showed signs of active denervation (6-year follow-up). Autonomic neuropathy has been reported in a case of post-polio syndrome (20). However, cerebellar symptoms as well as dementia cannot be explained by the diagnosis of post-polio syndrome.

### Case 3: 59–62 Years, Probable MSA-P

#### Clinical Phenotype

Two years of symmetric Parkinsonism (bradykinesia, rigidity, and postural instability), shuffling gait, postural instability, wheeled walker, impaired fine motor skills; *non-motor*: urinary retention requiring catheterization, obstipation, cold-hand sign, and severe neurogenic orthostatic dysregulation; UMSARS = 26 pts.

#### Imaging

*cardiac 123I-MIBG scintigraphy:* H/M ratio 1.45.

*123I-FP-CIT SPECT:* asymmetric (right) reduced tracer uptake (putamen, striatum, nucleus caudatus).

#### Discussion

Reduction of deep tendon reflexes (ankle) led initially to the suspicion of axonal motor neuropathy. However, protopathic sensibility and bathyesthesia were unremarkable, studies of compound nerve conduction velocity, somatosensible potentials as well as sympathetic skin response (upper extremities) were within normal limits. Thus, reduced cardiac 123I-MIBG binding might be related to clinically insignificant ganglionopathy.

### Case 4: 73–76 Years, Essential Tremor

#### Clinical Phenotype

3.5 year history of action and postural tremor (right > left), no bradykinesia or rigidity; *non-motor*: none (including autonomic history).

#### Imaging

*cardiac 123I-MIBG scintigraphy:* H/M ratio 1.49.

*123I-FP-CIT SPECT:* normal.

#### Discussion

Patient has a history of type II diabetes that led to distal symmetric neuropathy (hypopallesthesia, absent ankle deep tendon reflex) and sick-sinus syndrome. Reduction of cardiac 123I-MIBG binding has been described in both, sick-sinus-syndrome (21) and diabetic neuropathy (19). However, the effect of amiodarone is discussed controversially. On the one hand amiodarone is postulated to reduce 123I-MIBG uptake directly and on the other hand, through an improved sympathetic tone due to long-term administration, supposed to increase 123I-MIBG uptake and block partially 123I-MIBG washout (14). Due to long half-life amiodarone might not completely washed out prior to 123I-MIBG scintigraphy.

### Case 5: 53–56 Years, Parkinson's Disease

#### Clinical Phenotype

Two years of mild asymmetric Parkinsonism (bradykinesia, rigidity, disturbed fine motor skills), dysarthria, hypomimia; *non-motor*: hypersomnia, depressive mood; UPDRS = 18 pts.

#### Imaging

*cardiac 123I-MIBG scintigraphy:* H/M ratio 2.08.

*123I-FP-CIT SPECT:* asymmetric (left) reduced tracer uptake (striatum, putamen).

#### Discussion

Patients major complain was dysarthria (palilalia) that was unresponsive to levodopa. Subsequently, the treating neurologist recommended bilateral subthalamic nucleus stimulation that was ineffective for dysarthria and all other Parkinson symptoms. During the 8-year follow-up, patients' motor symptoms progressed and he developed supranuclear gaze palsy and severe postural instability. The patient did not develop autonomic failure and no frontal-executive dysfunction. Eventually, the diagnosis of Progressive Supranuclear Palsy (PSP—Parkinson phenotype, PSP rating scale = 24 pts., PSP staging system = 3) was established and deep brain stimulation was discontinued. In this case, cardiac 123I-MIBG scintigraphy correctly pointed toward non-idiopathic Parkinson's disease. Clinical phenotype (Parkinsonism), preserved levodopa response, and absence of gaze palsy prevented early recognition of PSP.

### Case 6: 64–67 Years, Parkinson's Disease

#### Clinical Phenotype

Two years history of asymmetric bradykinesia, rigidity and predominant resting tremor; *non-motor*: none, ARS without cardiovagal or adrenergic failure; UPDRS = 24 pts.

#### Imaging

*cardiac 123I-MIBG scintigraphy:* H/M ratio 2.11.

*123I-FP-CIT SPECT:* bilateral reduced tracer uptake (putamen, striatum, nucleus caudatus).

#### Discussion

No definite confounder of cardiac MIBG binding could be identified in this case. Patient's pharmacotherapy was limited to calcium channel blocker and rasagiline. Even though, calcium channel blockers might increase slightly H/M ratio, the extent of increased H/M ratio is not reasonable (14). Moreover, there is no evidence for rasagiline altering 123I-MIBG uptake. There were no clinical signs or symptoms if concomitant neuropathy and no history of unstable angina pectoris characteristics.

### Case 7: 80–83 Years, Parkinson's Disease

#### Clinical Phenotype

Four years of asymmetric (left) severe bradykinesia and rigidity, resting and postural tremor, postural imbalance, camptocormia; *non-motor*: none, no evidence of cardiovagal or adrenergic failure in ARS, no orthostatic hypotension; UPDRS = 29 pts.

#### Imaging

*cardiac 123I-MIBG SPECT:* H/M ratio 2.23, corrected to 2.16.

*123I-FP-CIT SPECT:* asymmetric (left) reduced tracer uptake (putamen, striatum, nucleus caudatus).

#### Discussion

Patient had a history of celiac disease and intestinal resection. Revision of the 123I-MIBG scintigraphy data revealed an erroneous position of the mediastinal ROI covering relocated stomach. Despite that correction, 123I-MIBG H/M ratio still lay within normal limits (2.16).

## Discussion

Myocardial 123I-MIBG uptake reflects the density and integrity of postganglionic sympathetic nerve endings. Due to the differential involvement of postganglionic sympathetic fibers in PD but not MSA, reduction of cardiac 123I-MIBG uptake is used to discriminate both disorders (6). While several studies (23, 24) propose an association of reduced 123I-MIBG H/M ratio in PD with longer disease duration, occurrence of non-motor symptoms and autonomic impairment, others (25, 26) could not confirm this relationship.

In our clinical series, indication for cardiac 123I-MIBG examination based on either atypical clinical course or symptom presentation. As such, our sample represent a highly selected but in clinical practice common group of patients, as nuclear medical exams are usually not ordered in clinical obvious cases. Among these patients, nuclear medical diagnostic classification based on cardiac 123I-MIBG scintigraphy differed from the clinically suspected syndrome in one third of cases. Furthermore, group wise comparison of cardiac 123I-MIBG H/M ratio failed in this small and selected sample to separate MSA from PD. This is unexpected considering the high sensitivity and specificity of cardiac 123I-MIBG scintigraphy to differentiate PD from other neurodegenerative disorders [pooled specificity range from 77 (8) to 91% (7)].

The discrepancy between nuclear medical and clinical diagnosis is explained in part by known confounders of 123I-MIBG scintigraphy. 123I-MIBG binding is reduced in areas with impaired sympathetic innervation such as fibrous tissue (e.g., myocardial infarction). However, none of our patients showed focal defects in myocardial 123I-MIBG uptake nor had a history of unstable angina pectoris characteristics. One PD patient (case 7) suffered from mild coronary heart disease without effect on H/M ratio. 123I-MIBG uptake is reduced when other compounds (14) compete for norepinephrine transporter binding at the presynaptic membrane of postganglionic sympathetic neurons. Moreover, besides this direct alteration of 123I-MIBG uptake, amiodarone is discussed to slightly increase 123I-MIBG uptake through improvement of sympathetic tone (case 4) (14). Cardiac autonomic neuropathy or ganglionopathy can cause cardiac sympathetic denervation and thus low 123I-MIBG binding (19). Despite being clinically unremarkable, ganglionopathy might underlie H/M ratio reduction in case 3. For a summary of the possible pitfalls in interpreting 123I-MIBG results, based on our clinical series, see Table 2.

More recent studies however, confirm our observation and report patients with unexpected results of cardiac 123I-MIBG scintigraphy, not sufficiently explained by concomitant medication or pathology. Nagayama et al. (11) reported on H/M ratio below the cut-off in 30 out of 96 cardiac 123I-MIBG examinations performed in 52 MSA patients. Neuropathological studies further indicate myocardial sympathetic involvement in MSA, leading probably to a decreased 123I-MIBG uptake. Orimo et al. revealed reduced TH-immunoreactivity in cardiac tissue and sympathetic ganglia of 6/15 MSA patients (27). Moreover, Sone et al. observed phosphorylated α-synuclein deposits as neuronal cytoplasmic inclusions in sympathetic ganglia of 11/26 MSA patients, that could be partially classified as Lewy bodies (hematoxylin-eosin staining) (28). Cardiac sympathetic denervation in MSA is unlikely the result of secondary postganglionic sympathetic degeneration as H/M ratio shows no temporal trend in repeated 123I-MIBG scans and H/M ratio does not correlate with clinical severity (11). Cook et al. (29) reported almost abolished norepinephrine content in myocardial tissue in an autopsied MSA patient with α-synuclein deposits limited to glia cells but not in neurons or sympathetic ganglia. Thus, minor cardiac sympathetic denervation does occur in MSA. In difference to PD, cardiac sympathetic degeneration in MSA is limited to TH-immunoreactive sympathetic nerve fibers (27) and an association with Lewy body pathology is still diversely discussed (28, 29).

With respect to PD, Orimo et al. (30) reported a pooled sensitivity of the delayed H/M ratio of 89.7% to detect Lewy body pathology. However, 123I-MIBG scintigraphy quantifies cardiac sympathetic denervation that is associated with Parkinsonism but not the underlying Lewy body pathology. It is noteworthy that in non-neurodegenerative Parkinsonism (associated with Parkin, DJ-1, PINK1, and LRRK2 mutations) cardiac sympathetic impairment is far more heterogeneous and 123I-MIBG scintigraphy remains unremarkably in over 50% of cases (23). Involvement of cardiac sympathetic fibers has been reported in early and premotor stages of PD (incidental Lewy body disease), even before neuronal cell loss and Lewy body pathology could be detected within the dorsal vagal nucleus and the nigrostriatal dopaminergic system (5). This may account for the reduced cardiac 123I-MIBG uptake in early stages of PD as reported by Umemura et al. (26). In the same study, however, 28 mostly early stage PD patients (15%) had H/M ratios above the cut-off [1.85 (26)]. In another prospective trial of 70 PD patients with 2 or more 123I-MIBG exams, mean H/M ratio was reduced at baseline and declined significantly over a 3-year follow-up (23). Nevertheless, 28/70 individuals had only mildly reduced or normal 123I-MIBG H/M ratios at baseline. Finally, Kim et al. (24) raise the question whether a “normal heart” phenotype of PD exists. In their study of 160 cases with *de-novo* PD, 44 had normal cardiac 123I-MIBG uptake.

In contrast to well-defined and highly selected samples in prospective trials, we report in our clinical series on the performance of cardiac 123I-MIBG in a clinical setting. As such, indication for nuclear imaging was based solely on individual clinical necessity, which in turn causes a selection bias toward non-typical symptom presentation and early disease stages whereas cases with instant clinical diagnosis are neglected. Among Parkinson patients however, age-dependent metabolic and vascular conditions are common. We deliberately did not exclude patients with concomitant conditions (other than history of unstable angina pectoris characteristics pointing to a probable myocardial infarction) that potentially involve the CNS or affect autonomic testing to assess feasibility of cardiac 123I-MIBG imaging under actual clinical conditions. As neuropathological confirmation is not available, final diagnostic classification relies on combined clinical criteria (comprehensive clinical exam, levodopa responsiveness, and clinical follow-up) of limited specificity as well.

In summary, cardiac 123I-MIBG scintigraphy is a highly specific and valuable tool to discriminate Parkinson syndromes and to access postganglionic autonomic (cardiac) impairment. 123I-MIBG analysis delivers important insights in pathophysiological processes and plays a significant role in scientific approaches and in specific clinical diagnostics in the broad spectrum of movement disorders and other diseases and syndromes. Group wise comparisons of delayed H/M ratio proved repeatedly to be sensitive and specific in detection and differentiation of PD from other neurodegenerative disorders. As in other imaging diagnostics, frequent pitfalls and restrictions have to be recognized and considered in the interpretation of these findings. We need to emphasize that 123I-MIBG scintigraphy is usually applied in geriatric patients, an age group where comorbidities and these pitfalls occur frequently. Thus, predictability for individual cases and feasibility in routine clinical practice are diminished. The confounding effects of concomitant medications, a progressive decline of 123I-MIBG uptake [~3%/year (23)] and a growing understanding of the complex and overlapping pattern of neurodegeneration in Parkinson syndromes, still requires clinical verification of the 123I-MIBG scintigraphy results on an individual basis. In the routine clinical practice, interpretation and drawing consequences from 123I-MIBG scintigraphy results have to be done carefully and should be in the hands of very experienced physicians and movement disorder specialists, respectively.

## Data Availability

The raw data supporting the conclusions of this manuscript will be made available by the authors, without undue reservation, to any qualified researcher.

## Author Contributions

AL and CS contributed conception and design of the study. LZ organized the database and performed the statistical analysis. CS wrote the first draft of the manuscript. AL, LZ, and CS wrote sections of the manuscript. All authors contributed to manuscript revision, read and approved the submitted version.

### Conflict of Interest Statement

The authors declare that the research was conducted in the absence of any commercial or financial relationships that could be construed as a potential conflict of interest.
