# Supplementary material for: Cardiac 123I-MIBG Scintigraphy in Neurodegenerative Parkinson Syndromes: Performance and Pitfalls in Clinical Practice
Source: Front Neurol. 2019 Feb 26;10:152. doi: 10.3389/fneur.2019.00152 (PMC6399127; doi:10.3389/fneur.2019.00152)
Supplement: Supplementary file 1 [file Data_Sheet_1.pdf]

Supplementary Material

Cardiac 123I-MIBG scintigraphy in neurodegenerative Parkinson syndromes: performance and pitfalls in clinical practice

Cornelia Skowronek*, Leonora Zange, Axel Lipp

*** Correspondence:**
Cornelia Skowronek, MD
cornelia.skowronek@charite.de

Detailed case descriptions

Case 1: 59-62 yrs., possible MSA-P

Clinical vignette

first symptoms: bradykinesia and rest tremor pronounced at left side

clinical phenotype (5 yrs.): symmetric bradykinesia and rigidity with postural instability and shuffling gait; startle myoclonus and pyramidal tract signs (hyperreflexia) within the left upper extremity; *non-motor*: severe palilalia and dysphagia, urge incontinence, obstipation, erectile dysfunction, sialorrhea and mild neurogenic orthostatic hypotension; UMSARS = 38 pts.

levodopa responsiveness: none up to 1200 mg/day

medical history: essential tremor

medication: amantadine 300 mg, propranolol 40 mg, clonazepam 0.5 mg, macrogol 1 sachet

Imaging

cardiac 123I-MIBG scintigraphy: H/M ratio 1.27

123I-FP-CIT SPECT: visually asymmetric (right) reduced putaminal tracer uptake

123I-IBZM SPECT: reduced postsynaptic D2 receptor density right > left

transcranial mesencephalic sonography: normal echogenicity of substantia nigra

cranial MRI: no structural lesions within the basal ganglia, no atrophy

Clinical follow-up: 9.2 yrs. Progression of motor symptoms and severe autonomic symptoms (pandysautonomia) until death 4 yrs. after recruitment.

follow-up diagnosis: probable MSA-P

Discussion: Clinical follow up (rapid progression, early death) strengthened the initial diagnosis of MSA-P. Medical history and clinical exam did not reveal clinically significant neuropathy nor concomitant medication sufficiently explaining reduced cardiac 123I-MIBG uptake.

Case 2: 63-66 yrs., possible MSA-C

Clinical vignette

first symptoms: blurred vision, gait imbalance, disturbed fine motor skills

clinical phenotype (5 yrs.): right sided Parkinsonism (bradykinesia, rigidity), pancerebellar syndrome and pyramidal tract signs; *non-motor*: apraxia, dysphagia, obstipation, erectile dysfunction, sialorrhea and mild neurogenic orthostatic hypotension; UMSARS = 33 pts.

levodopa responsiveness: none up to 1300 mg/day

medical history: poliomyelitis in childhood

medication: amantadine 100 mg, levodopa/benserazide 900/100mg

Imaging

cardiac 123I-MIBG scintigraphy: H/M ratio 1.14

123I-FP-CIT SPECT: visually symmetric reduced tracer uptake (putamen, striatum, nucleus caudatus)

123I-IBZM SPECT: bilateral normal postsynaptic D2 receptor density

transcranial mesencephalic sonography: normal echogenicity of substantia nigra

cranial MRI: mild vascular leukoencephalopathy, mild temporal and cerebellar atrophy, with no significant progression within 6 yrs.

Clinical follow-up: 11.2 yrs.; progression of cerebellar syndrome, pyramidal tract signs, mutism, pandysautonomia and severe cognitive impairment (mnestic deficits, mini-mental state examination = 19/30 pts.).

follow-up diagnosis: possible MSA-C

Discussion: Clinical diagnosis in this case is less robust as current consensus diagnostic criteria consider dementia as a non-supporting feature of MSA. However, MODIMSA study (19) showed evidence that frontalexecutive dysfunction and cognitive impairment is indeed associated with MSA. Spinocerebellar atrophy (SCA) causes progressive ataxia, Parkinsonism and pyramidal tract signs but patients' familiar history was unremarkable and genetic screen for SCA 1,2,3 and 6 was negative. Patient had a history of poliomyelitis during childhood and thus might have developed post-polio syndrome. However, lower motoneuron disability in our patient did not progress clinically nor does electromyography showed signs of active denervation (6-year follow-up). Autonomic neuropathy has been reported in a case of post-polio syndrome (20). However, cerebellar symptoms as well as dementia cannot be explained by the diagnosis of post-polio syndrome.

Case 3: 59-62 yrs., probable MSA-P

Clinical vignette

first symptoms: left sided bradykinesia, shuffling gait, falls, urge incontinence

clinical phenotype (2 yrs.): symmetric Parkinsonism (bradykinesia, rigidity and postural instability), shuffling gait, postural instability, wheeled walker, impaired fine motor skills; *non-motor*: urinary retention requiring catheterization, obstipation, cold-hand sign and severe neurogenic orthostatic dysregulation; UMSARS = 26 pts.

levodopa responsiveness: limited reduction of rigidity with 1000 mg/day

medical history: depressive episodes

medication: none

Imaging:

cardiac 123I-MIBG scintigraphy: H/M ratio of 1.45

123I-FP-CIT SPECT: asymmetric (right) reduced tracer uptake (putamen, striatum, nucleus caudatus)

cranial MRI: no ischemic lesions in basal ganglia, no regional atrophy, no putaminal rim sign or hypodensity, respectively

Clinical follow-up: 3.3 yrs.; rapid progression of gait impairment (single steps with two helpers) and increasing muscle tone and developing autonomic pandysautonomia.

follow-up diagnosis: probable MSA-P

Discussion: Reduction of deep tendon reflexes (ankle) led initially to the suspicion of axonal motor neuropathy. However, protopathic sensibility and bathyesthesia were unremarkable, studies of compound nerve conduction velocity, somatosensible potentials as well as sympathetic skin response (upper extremities) were within normal limits. Thus, reduced cardiac MIBG binding might be related to clinically insignificant ganglionopathy.

Case 4: 73-76 yrs., Essential Tremor

Clinical vignette

first symptoms: tremor of upper limbs (right > left)

clinical phenotype (3.5 yrs.): action and postural tremor (right > left), no bradykinesia or rigiditiy; *non-motor*: none (including autonomic history)

levodopa responsiveness: none up to 200 mg/day

medical history: arterial hypertension, hyperlipoproteinemia, sick sinus syndrome, pacemaker

medication: metixen 7.5 mg, propranolol 80 mg, amiodarone 200 mg, enalapril 20 mg, hydrochlorothiazide 25 mg, metformin 1275 mg, simvastatin 20 mg, phenprocoumon 1.5 - 3 mg

Imaging

cardiac 123I-MIBG scintigraphy: H/M ratio of 1.49

123I-FP-CIT SPECT: normal tracer uptake

transcranial mesencephalic sonography: normal echogenicity of substantia nigra

cranial CT Scan: no signs of specific brain atrophy, no vascular lesions within basal ganglia

Clinical follow-up: 6.8 yrs.; minor progression of postural and resting tremor, no additional clinical signs of Parkinsonism such as bradykinesia, rigidity or postural instability.

follow-up diagnosis: Essential Tremor

Discussion: Patient has a history of type II diabetes that led to distal symmetric neuropathy (pallhypaethesia, absent ankle deep tendon reflex) and sick-sinus syndrome. Reduction of cardiac 123I-MIBG binding has been described in both, sick-sinus-syndrome (21) and diabetic neuropathy (22). However, the effect of amiodarone is discussed controversially. On the one hand amiodarone is postulated to reduce 123I-MIBG uptake directly and on the other hand, through an improved sympathetic tone, supposed to increase 123I-MIBG uptake and block partially 123I-MIBG washout (14) and due to long half-life amiodarone might not completely washed out prior to 123I-MIBG scintigraphy.

Case 5: 53-56 yrs., Parkinson's Disease

Clinical vignette

first symptoms: bradykinesia

clinical phenotype (2 yrs.): mild asymmetric Parkinsonism (bradykinesia, rigidity, disturbed fine motor skills),

dysarthria, hypomimia; *non-motor*: hypersomnia, depressive mood; UPDRS = 18 pts.

levodopa responsiveness: limited motor response (UPDRS off = 18 pts. / on = 12 pts.)

medical history: lumbar disc prolapse

medication: ropinirole ret. 20 mg, rasagaline 1 mg

Imaging

cardiac 123I-MIBG scintigraphy: H/M ratio 2.08

123I-FP-CIT SPECT: asymmetric (left) reduced tracer uptake (striatum, putamen)

transcranial mesencephalic sonography: bilateral hyperechogenicity of substantia nigra

cranial MRI: no atrophy or ischemic lesions in basal ganglia or cerebellum

Clinical follow-up: 8.6 yrs.; loss of levodopa responsiveness, severe palilalia, supranuclear gaze palsy.

follow-up diagnosis: Progressive Supranuclear Palsy (PSP)

Discussion: Patients major complain was dysarthria (palialia) that was unresponsive to levodopa. Subsequently, the treating neurologist recommended bilateral subthalamic nucleus stimulation that was ineffective for dysarthria and all other Parkinson symptoms. During the 8-year follow-up, patients' motor symptoms progressed and he developed supranuclear gaze palsy and severe postural instability. The patient did not develop autonomic failure and no frontal-executive dysfunction. Eventually, the diagnosis of Progressive Supranuclear Palsy (PSP - Parkinson phenotype, PSP rating scale = 24 pts., PSP staging system = 3) was established and deep brain stimulation was discontinued. In this case,

cardiac 123I-MIBG scintigraphy correctly pointed towards non idiopathic Parkinson's Disease. Clinical phenotype (Parkinsonism), preserved levodopa response and missing gaze palsy prevented early recognition of PSP.

Case 6: 64-67 yrs., Parkinson's Disease

Clinical vignette

first symptoms: tremor right hand

clinical phenotype (2 yrs.): asymmetric bradykinesia, rigidity and resting > postural tremor; *non-motor*: none, ARS without cardiovagal or adrenergic failure; UPDRS = 24 pts.

levodopa responsiveness: sufficient tremor and rigidity reduction (600 mg/d)

medical history: migraine, arterial hypertension

medication: rasagaline 1 mg, nisoldipine 20 mg

Imaging

cardiac 123I-MIBG scintigraphy: H/M ratio of 2.11

123I-FP-CIT SPECT: bilateral reduced tracer uptake (putamen, striatum, nucleus caudatus)

transcranial mesencephalic sonography: normal echogenicity of substantia nigra

cranial MRI: no structural lesions in basal ganglia, no atrophy

Clinical follow-up: 6.7 yrs.; progressive hypokinetic-rigid syndrome with sustained dopaminergic responsiveness.

follow-up diagnosis: Parkinson's Disease

Discussion: No definite confounder of cardiac MIBG binding could be identified in this case. Patient's pharmacotherapy was limited to calcium channel blocker and rasagiline. Even though, calcium channel blockers might increase slightly H/M ratio, the extent of increased H/M ratio is not reasonable (14). Moreover, there is no evidence for rasagiline altering 123I-MIBG uptake. that could reduce H/M ratio but not increase. There were no clinical signs or symptoms if concomitant neuropathy and no history of unstable angina pectoris characteristics.

Case 7: 80-83 yrs., Parkinson's Disease

Clinical vignette

first symptoms: bradykinesia, tremor, gait imbalance

clinical phenotype (4 yrs.): asymmetric (left) severe bradykinesia and rigidity, resting and postural tremor, postural imbalance, camptocormia; *non-motor*: none, no evidence of cardiovagal and adrenergic failure in ARS, no orthostatic hypotension; UPDRS = 29 pts.

levodopa responsiveness: > 30% with 1000 mg/d (UPDRS off = 43 pts. / on = 29 pts.)

medical history: coronary heart disease, heart insufficiency (NYHA II), arterial hypertension, celiac disease with intestinal resection
medication: levodopa/benserazide 1000/250 mg, ramipril 2.5 mg, bisoprolol 5 mg, vitamin C 1000 mg

Imaging

cardiac 123I-MIBG scintigraphy: H/M ratio of 2.23, corrected to 2.16

123I-FP-CIT SPECT: asymmetric (left) reduced tracer uptake (putamen, striatum, nucleus caudatus)

transcranial mesencephalic sonography: hyperechogenicity of right substantia nigra

cranial MRI: leucencephalopathy, global atrophy, no lesions within basal ganglia

Clinical follow-up: 4.9 yrs.; moderate progression of Parkinson related motor symptoms with sustained dopaminergic responsiveness.

follow-up diagnosis: Parkinson's Disease

Discussion: Patient had a history of celiac disease and intestinal resection. Revision of the 123I-MIBG scintigraphy data revealed an erroneous position of the mediastinal ROI covering relocated stomach. Despite that correction, 123I-MIBG H/M ratio still lay within normal limits (2.16).
